# Angucycline-like Aromatic Polyketide from a Novel *Streptomyces* Species Reveals Freshwater Snail *Physa acuta* as Underexplored Reservoir for Antibiotic-Producing Actinomycetes

**DOI:** 10.3390/antibiotics10010022

**Published:** 2020-12-29

**Authors:** Nasim Safaei, Yvonne Mast, Michael Steinert, Katharina Huber, Boyke Bunk, Joachim Wink

**Affiliations:** 1 Helmholtz Centre for Infection Research, Department of microbial Strain Collection, Inhoffenstrasse 7, D-38124 Braunschweig, Germany; Nasim.safaei@helmholtz-hzi.de; 2 German Center for Infection Research (DZIF), Leibniz Institute DSMZ, Inhoffenstrasse 7, D-38124 Braunschweig, Germany; Yvonne.mast@dsmz.de (Y.M.); Katharina.huber@dsmz.de (K.H.); Boyke.bunk@dsmz.de (B.B.); 3 Technical University of Braunschweig, Spielmannstr. 7, 38106 Braunschweig, Germany; m.steinert@tu-bs.de

**Keywords:** Actinobacteria, *Physa acuta*, *Streptomyces*, antibiotics, angucycline

## Abstract

**Simple Summary:**

The current study shows that freshwater snails can be considered as new sources for bioactive metabolites, since a novel *Streptomyces* species 7NS3 produced four active compounds against Gram-positive bacteria. One of the compounds was an angucycline-like aromatic polyketide matched with a known compound, emycin A. Genome mining studies based on the whole-genome sequence of 7NS3 resulted in the identification of a gene cluster potentially coding for emycin A biosynthesis.

**Abstract:**

Antibiotic producers have mainly been isolated from soil, which often has led to the rediscovery of known compounds. In this study, we identified the freshwater snail *Physa acuta* as an unexplored source for new antibiotic producers. The bacterial diversity associated with the snail was characterized by a metagenomic approach using cultivation-independent high-throughput sequencing. Although Actinobacteria represented only 2% of the bacterial community, the focus was laid on the isolation of the genus *Streptomyces* due to its potential to produce antibiotics. Three *Streptomyces* strains (7NS1, 7NS2 and 7NS3) were isolated from *P. acuta*, and the antimicrobial activity of the crude extracts were tested against a selection of Gram-positive and Gram-negative bacteria and fungi. 7NS3 showed the strongest activity against Gram-positive bacteria and, thus, was selected for genome sequencing and a phylogenomic analysis. 7NS3 represents a novel *Streptomyces* species, which was deposited as *Streptomyces* sp. DSM 110735 at the Leibniz Institute-German Collection of Microorganisms and Cell Cultures (DSMZ). Bioassay-guided high-performance liquid chromatography (HPLC) and high-resolution electrospray ionization-mass spectrometry (HR-ESI-MS) analyses of crude extract fractions resulted in the detection of four compounds, one of which matched the compound characteristics of emycin A, an angucycline-like aromatic polyketide. Genome mining studies based on the whole-genome sequence of 7NS3 resulted in the identification of a gene cluster potentially coding for emycin A biosynthesis. Our study demonstrates that freshwater snails like *P. acuta* can represent promising reservoirs for the isolation of new antibiotic-producing actinobacterial species.

## 1. Introduction

Actinobacteria are one of the largest bacterial phyla in terms of the variety of identified species [1]. About 45% of all bioactive microbial metabolites are produced by filamentous actinomycetales species [2]. More than 95% of all actinomycetales strains isolated from the soil belong to the genus *Streptomyces* [3]. Streptomycetes are the most important source for antibiotics, since they produce up to 75% of all clinically used antibiotics [4].

Due to a steady decline in new compound discoveries from terrestrial microbial sources, there is a growing interest to explore under-investigated habitats [5]. Such underexplored habitats are, for example, represented by natural symbiotic ecosystems. The antibiotics produced from Actinobacteria in a multilateral symbiosis system are tractable for functional and evolutionary analyses [6]. One of the widely studied mutualistic symbioses is evident in insect-associated Actinobacteria, as exemplified by the European beewolf that harbors the antibiotic-producing “*Candidatus* Streptomyces philanthi” in antennal glands to protect wasp larvae from fungal infections [7]. The mutualistic *Streptomyces* incorporated into beewolf cocoons provide a combination of nine antibiotic substances, including streptochlorin and a complex of eight piericidin derivatives, which are expected to protect the host against the fungal pathogen. In return, the host provides nutrition and a competition-free ecological niche for the bacterium [6,8]. Another example of symbiotic association for protection of the host against antagonistic fungi is observed in the beetle *Dendroctonus frontalis* [9]. The bioactive compound mycangimycin, produced by the bacterial symbiont *Streptomyces* sp. SPB74, prevents the growth of *Ophiostoma minus,* which is the beetle’s fungal antagonist [10].

After insects, Mollusca with over 90,000 extant described species is the second-largest phylum of invertebrates [9,10]. Although molluscan species are mostly inhabitants of oceans, and oceans cover 70% of the Earth’s surface, less than 1% of them have been investigated for secondary metabolites [11,12]. Gastropods, including snails, are the largest group of mollusks [13]. Snail-associated Actinobacteria, were investigated by Peraud et al. in 2009 for the first time. From 57 isolated actinomycetes, 38% accounted for *Streptomyces* morphotypes [14]. Streptomycetes are well-known for their antibiotic-producing potential. Especially new species within the genus *Streptomyces* from unique habitats, as, for instance, from mollusks, are promising in terms of drug discovery [14]. For example, *Streptomyces* sp. CP32 was isolated as a new strain from the cone snail *Conus pulicarius* and produces the neuroactive thiazoline metabolites pulicatins A-E [15]. Likewise, totopotensamides A and B were extracted and identified from *Streptomyces* sp. 1053U.I.1a.3b isolated from the gastropod mollusk *Lienardia totopotens* [16].

Freshwater snails are another underexplored habitat for potential antibiotic-producing species. They show a ubiquitous distribution in freshwater aquatic habitats like rivers, swamps, lakes, ponds and springs [17]. Freshwater snails are the main intermediate host for over 10,000 species of trematodes [18]. There is proof that the intestinal microbiota is not just an indiscriminate set of microorganisms but, rather, a selective community that plays a crucial role in the metabolism, growth, digestion and immune system of the host [17,18,19]. It has been hypothesized that the microbiota of freshwater snails provides important implications of the immune system of the host, preventing invasion by exogenous pathogenic microbes such as parasites [10,16]. *Physa acuta* (Draparnaud, 1805) (syn. *Physella acuta*) is well-known as a globally invasive freshwater snail [18] that acts as a host for trematodes, causing diseases like echinostomiasis and fascioliasis [20]. So far, freshwater snails have not been targeted as a source for isolating new species that may contain new natural compounds but, mostly, as a promising source for novel enzymes [19].

Based on metagenomics as a culture-independent approach, it has been found that the majority of bacteria remains nonculturable when using standard laboratory cultivation approaches [2,21]. Although genomics tools nowadays allow to access microbial diversity, still, the cultivation of new bacterial candidates is essential for biotechnological purposes [22].

In this study, we evaluated the bacterial community composition of *P. acuta.* We focused on isolating new species of the genus *Streptomyces* in the family *Streptomycetaceae* within the phylum Actinobacteria as well-known natural compound producers. The *Streptomyces* sp. 7NS3 (7NS3) showed good antibacterial activity, especially against Gram-positive bacteria. The phylogenetic analysis based on the 16S rRNA gene and whole-genome sequence analysis suggest that 7NS3 represents a novel *Streptomyces* species, which was deposited as *Streptomyces* sp. DSM 110735 at the German Collection of Microorganisms and Cell Cultures (DSMZ) strain collection. HR-ESI-MS (high-resolution electrospray ionization-mass spectrometry) analysis of the 7NS3 culture extracts revealed the presence of an angucycline-like substance similar to emycin A. Genome mining of 7NS3 led to the identification of a type II polyketide biosynthetic gene cluster potentially encoding for the emycin A substance. Our work indicates that freshwater snails represent an untapped reservoir for novel antibiotic-producing species and illustrates the potential of the combination of cultivation methods and culture-independent approaches in natural product discovery.

## 2. Results

### 2.1. The Physa acuta Microbiome and Distribution of Actinobacterial Families

**Figure 1:**
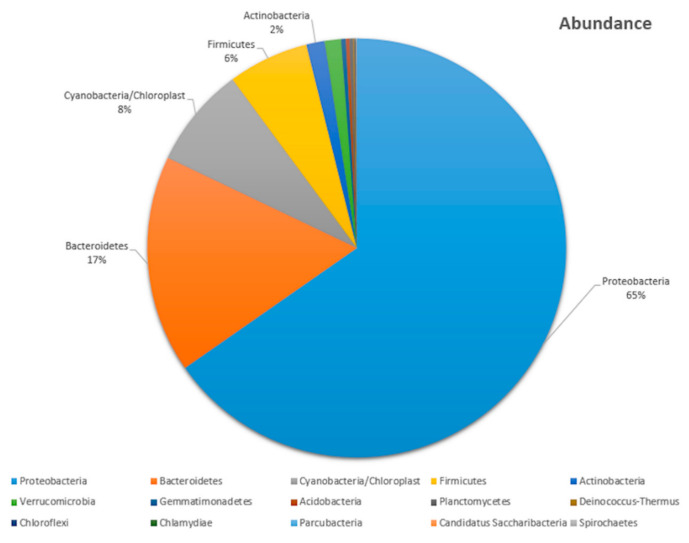
Bacterial taxonomic profile of the Physa acuta microbiome at the phylum level. Proteobacteria was the most predominant phylum (65% abundance), followed by Bacteroidetes (17%), Cyanobacteria/Chloroplast (8%), Firmicutes (6%) and Actinobacteria (2%).

To gain insight into the composition of the bacterial community, especially of the phylum Actinobacteria, a metagenome approach was applied, whereby amplicons of the V3 region of the 16S rRNA gene were analyzed. Amplicon sequencing yielded 340 Mbp of raw sequence data in the form of 1,124,851 paired-end reads, with read lengths of 2 × 151 bp. After the amplicon analysis, including the trimming, joining and removal of low-quality sequences, 894,391 full-length amplicons were obtained, from which 880,108 could be assigned to the bacterial domain by an**** Ribosomal Database Project (RDP) Classifier. The amplicon reads were grouped into 21 phyla. The predominant phyla were Proteobacteria (65%) and Bacteroidetes (17%), followed by Cyanobacteria and Chloroplast (8%) and Firmicutes (6%) (Figure 1). Actinobacteria make up 2% of the total bacterial population. Among 30 families of Actinobacteria, a total of 56 genera were identified. The most abundant actinobacterial families were *Kineosporiaceae* (26%, *n* = 1688), *Microbacteriaceae* (20%, *n* = 1298), *Acidimicrobiaceae* (18%, *n* = 1154) and *Nocardioidaceae* (10%, *n* = 622), where “*n*” represents the number of sequences identified within the respective families. There were four sequences that were identified as representatives of the family *Streptomycetaceae* (Table S1).

### 2.2. Isolation and Phylogenetic Analysis of a Snail-Associated Streptomyces Strain

**Figure 2:**
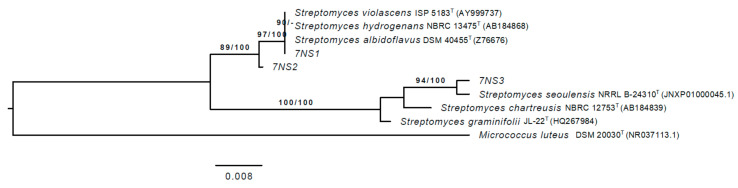
Maximum-likelihood phylogenetic tree based on the full-length 16S rRNA gene sequence of 7NS1, 7NS2 and 7NS3 and most related species. Numbers above the branches are bootstrap support values greater than 60% for the maximum-likelihood (left) and maximum-parsimony (right) methods. Bar: 0.008 (8 nucleotide substitution per 1000 nucleotides).

To investigate the potential of freshwater snails as a reservoir for the isolation of new antibiotic-producing species, we aimed to isolate Streptomycetes from the *P. acuta* microbiome. Three members of the genus *Streptomyces* were isolated, which were designated 7NS1, 7NS2 and 7NS3. Based on the 16S rRNA gene sequence comparisons, strain 7NS1 showed the closest phylogenetic similarity of 99.75% with the type strain *Streptomyces violascens* DSM 40183^T^, 7NS2 with *Streptomyces hydrogenans* FHP 678 (99.54%) and 7NS3 with *Streptomyces seoulensis* NRRL B-24310^T^ (99.59%). Based on the phylogenetic tree, the strains 7NS1 and 7NS2 belong to a different branch compared to strain 7NS3 (Figure 2). 7NS1 and 7NS2 were closely related to *S. violascens* DSM 40183^T^, *S. hydrogenans* FHP 678 and *Streptomyces albidoflavus* DSM 40455^T^, whereas 7NS3 showed the highest similarity to *S. seoulensis* NRRL B-24310^T^ (Figure 2).

### 2.3. Evaluation of Antimicrobial Activity Based on Minimum Inhibitory Concentration

The ability of producing antimicrobial compounds against nine microorganisms, including Gram-positive (*Staphylococcus aureus* (Newman), *Bacillus subtilis* DSM 10^T^ and *Mycobacterium smegmatis* ATCC 700084) and Gram-negative bacteria (*Escherichia coli* DSM 1116, *Pseudomonas aeruginosa* PA14 and *Citrobacter freundii* DSM 30039^T^), as well as yeast and fungi (*Candida albicans* DSM 1665, *Pichia anomala* DSM 6766 and *Mucor hiemalis* DSM 2656^T^), was tested by the minimum inhibitory concentration (MIC) assay. Crude extracts of 7NS1, 7NS2 and 7NS3 were obtained by cultivating the strains in cultivation medium 5294. The results of the antibacterial activity tests with the crude extracts showed that all the samples were active against all tested Gram-positive bacteria and the fungus *M. hiemalis* DSM 2656^T^. *S. aureus* (Newman) and *Bacillus subtilis* DSM 10^T^ were more strongly inhibited by crude extracts from 7NS2 and 7NS3. The highest activity was observed for the crude extract of strain 7NS3, with an MIC value of 8.3 µg/mL and 0.52 µg/mL against *S. aureus* (Newman) and *B. subtilis* DSM 10^T^, respectively (Table 1).

### 2.4. Chemical Analysis of Crude Extract from Strain 7NS3

**Figure 3:**
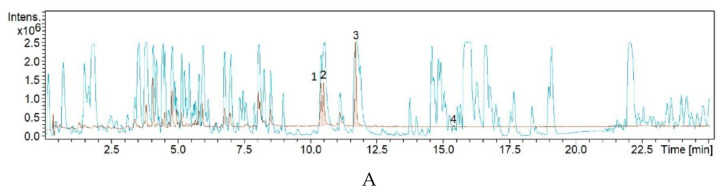

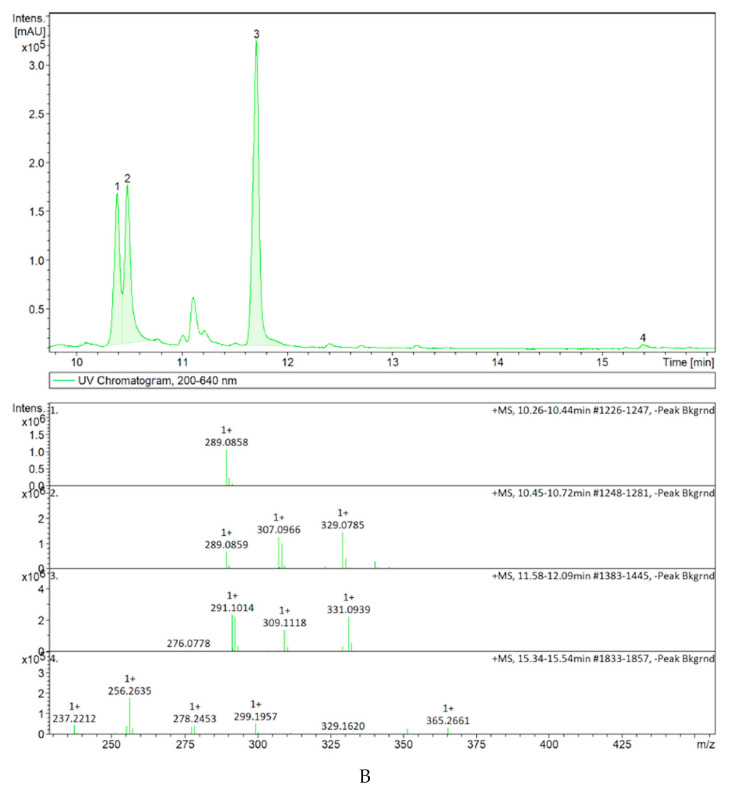
(**A**) High-resolution electrospray ionization-mass spectrometry (HR-ESI-MS) total ion current (TIC) of the 7NS3 crude extract. (**B**) Extracted ion chromatogram 200–640 nm for peaks 1–4. 1: *m*/*z* 289.0858MH^+^, retention time (t_R_) = 10.39 min, 2: *m*/*z* 307.0966 MH^+^, *m*/*z* 329.0785 MNa^+^, *m*/*z* 289.0859 (MH^+^–H_2_O), t_R_ = 10.49 min, 3: *m*/*z* 309.1118 MH^+^, *m*/*z* 331.0939 MNa^+^, *m*/*z* 291.1014 (MH^+^–H_2_O), t_R_ = 11.71 min and 4: *m*/*z* 256.2635 MH^+^, *m*/*z* 278.2453 MNa^+^, *m*/*z* 237.2212 (MH^+^–H_2_O), t_R_ = 15.39 min.

The crude extract of strain 7NS3 showed higher antimicrobial activity compared to the crude extracts of 7NS1 and 7NS2. The 7NS3 crude extract was fractionated by high-performance liquid chromatography (HPLC) and tested in bioassays against *B. subtilis* DSM 10^T^ and *S. aureus* Newman in order to determine the peak-related activities. The micro-fractionation was performed in 96-well plates to target the active compounds of the crude extract. The HPLC fractionation experiments resulted in four fractions that inhibited the growth of *B. subtilis* DSM 10^T^ and two fractions inhibiting *S. aureus* Newman (Figure S1A,B). In order to identify the associated target masses of the detected active fractions, the crude extract was analyzed by high-resolution electrospray ionization-mass spectrometry (HR-ESI-MS). The four peaks, which were correlated to activity in the HPLC chromatogram, were detected in the HR-ESI-MS total ion current (TIC) and named compounds **1** to **4** (Figure 3A). The mass spectra of compounds **1** with the retention time (t_R_) = 10.39 min showed ions at a mass-to-charge ratio (*m/z*) of 289.0857, corresponding to MH^+^ (Figure S2A). The UV/Vis absorption bands were located at 232 nm, 380 nm and 458 nm. The second compound, with a chromatographic retention time of 10.49 min, presented mass spectra with ions *m/z* 307.0966, *m/z* 329.0785 and *m/z* 289.0859 corresponding to MH^+^, MNa^+^ and (MH^+^ − H_2_O), respectively. The third molecule, with t_R_ = 11.49 min, showed ions *m/z* 309.1118, *m/z* 331.0939 and *m/z* 291.1014 corresponding to MH^+^, MNa^+^ and (MH^+^ − H_2_O), respectively. The suggested formulas of C_19_H_14_O_4_ and C_19_H_16_O_4_ were obtained for compounds **3** and **4** by the SmartFormula™ algorithm in Compass software from Bruker. A search in the Dictionary of Natural Products (DNP) database revealed that the protonated ion *m/z* 309.1118 MH^+^ matched with the known compound, emycin A. Emycin A has a molecular weight of 308.3436 g mol^−1^ (*m/z* 309.3436 MH^+^) and a formula of C_19_H_16_O_4_. The UV/Vis spectrum shows absorption maxima at 217 nm, 260 nm and 402 nm (Figure S3). The characteristic UV/Vis absorption bands at 220 nm, 260 nm and 402 nm of compounds **3** matched with those of emycin A (Figure S2C). The chromatogram pattern of compounds **1** and **2** indicated the structural isomers of the main compound. From the UV bands and mass similarity, it can be concluded that compounds **3** has a double bond compared to compounds **2**. Compounds **4** with a t_R_ of 15.39 min presents a mass spectra with ions *m/z* 255.2319 and *m/z* 256.2635 corresponding to MH^+^ that matched with hexadecenoic acid in the in-house Myxobase database. This fatty acid with the molecular formula of C_16_H_30_O_2_ has antibacterial activity against Gram-positive bacteria.

### 2.5. Genome Sequencing and Phylogenomic Analysis with TYGS

**Figure 4:**
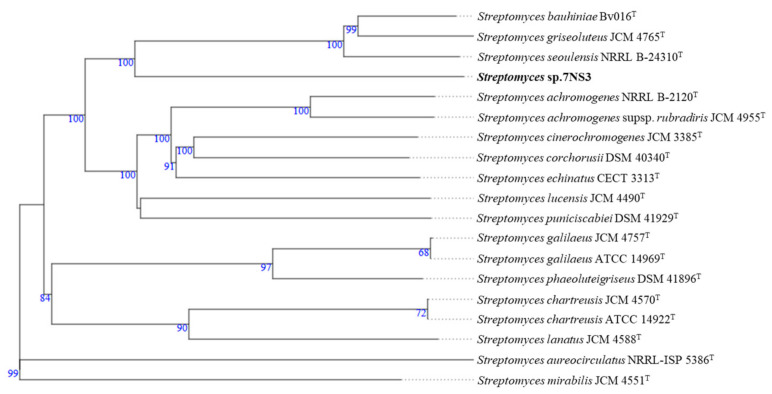
Whole-genome sequence tree generated with the TYGS web server for strain 7NS3 and its closely related species. The numbers above the branches are GBDP (Genome-BLAST Distance Phylogeny) pseudo-bootstrap support values >60% from 100 replications, with an average branch support of 84.4%.

Genomic studies were performed on 7NS3, and the draft genome sequence of 7NS3 was obtained by Illumina whole-genome sequencing. The genome was assembled to 49 quality-controlled contigs (>450 bp) summing up to a total genome size of 6.85 Mbp with a GC content of 71.6%. Digital DNA–DNA hybridization (dDDH) of 7NS3 and *S. seoulensis* NRRL B-24310^T^ showed a value of 29.5%, which was below the 70% threshold [23], suggesting that 7NS3 represents a novel species. A whole-genome phylogenetic analysis was performed with the TYGS server (https://tygs.dsmz.de/) [24] and revealed that 7NS3 shows the highest similarity to *S. seoulensis* NRRL B-24310^T^, which is located on a more distantly related branch (Figure 4).

### 2.6. Identification of Biosynthetic Gene Clusters for Strain 7NS3

In order to identify the potential secondary metabolite biosynthesis gene clusters (BGCs) of strain 7NS3, the genome sequence was analyzed with the bioinformatic tool antiSMASH 5.0. A total of 19 putative BGCs were detected, whereby six of them matched the known clusters for alkylresorcinol, scabichelin, informatipeptin, geosmin, albaflavenone and ectoine with 100% similarity. One BGC showed >80% similarity to a BGC encoding a potential spore pigment. Three BGCs showed >60% similarity to clusters encoding desferrioxamin, melanin and hopene. The remaining BGCs with similarities <50% were predicted to encode the biosynthetic machinery for the synthesis of two nonribosomal peptides, one polyketide, one polyketide-nonribosomal peptide hybrid, one thiopeptide, one siderophore, one lassopeptide, one terpene and one bacteriocin (Figure S4).

### 2.7. Analysis of the Type II Polyketide BGC

The largest family of polycyclic aromatic polyketides is the family of angucyclines, with various biological activities [25]. As outlined above, strain 7NS3 was found to produce an emycin A substance that belongs to the angucycline group. Gene cluster region 6.1, detected by an antiSMASH analysis, harbors a predicted type II polyketide BGC, which shows similarity to several known angucycline-type BGCs, such as the kiamycin BGC from *Streptomyces* sp. W007 (45% similarity) or the lugdunomycin BGC from *Streptomyces* sp. QL37 (44% similarity) [26,27]. The most similar genetic organization regarding the potential 7NS3 type II polyketide BGC genes was found to be present in strain *Streptomyces* sp. 303MFCol5.2 (PRJNA187949), for which a set of 26 genes (BGC region 24.1) showed 65% similarity to gene cluster region 6.1 of 7NS3. However, no type II polyketide has been described for *S.* sp. 303MFCol5.2 so far. A manual cluster analysis by using BLASTP on each individual gene of region 6.1 from 7NS3 revealed that only the gene region from nucleotide position 182,328–210,753 nt shows similarity to the genes of *S.* sp. 303MFCol5.2, whereas the up- and downstream regions have higher overall similarity to genes of strain *Streptomyces* sp. LaPpAH-108 (Figure S5). These gene similarity characteristics can be used as an indicator to define cluster-boundaries of the emycin A encoding cluster (herewith denominated *ang*) in 7NS3. Thus, we suggest that the *ang* gene cluster from 7NS3 covers the gene region 182,328–210,753 nt from locus tag IF665_12470-IF665_12600 (Table S2). The *ang* cluster harbors, overall, 28 open reading frames (orfs), which cover a size of ~28 kb. The clusters include the orfs IF655_12525 and IF655_12530 encoding a typical type II minimal polyketide synthase (PKS) system, as well as the orfs IF655_12520, IF655_12530 and IF655_12535 encoding a putative type II polyketide cyclase, a 3-oxoacyl-ACP reductase and an aromatase/cyclase, respectively. The predicted gene products are usually involved in angucycline-specific biosynthetic reactions, suggesting that they might be involved in the biosynthesis of the angucycline-like substance. 

## 3. Discussion

Within the phylum Actinobacteria, the genus *Streptomyces* is a well-known and promising producer of antibacterial compounds [14]. Many plants, insects and marine animals, including gastropods, benefit from *Streptomyces*-produced antibiotics to protect themselves against pathogens [28]. For instance, Polycyclic Tetramate Macrolactams (PTMs) are produced by cone snail-associated streptomycetes and protect marine snails of the genus *Conus* from fungal pathogens [29]. Freshwater snails are known as intermediate hosts for parasitic trematodes. To the best of our knowledge, these organisms have not been targeted as sampling sites for antibiotic-producing bacteria so far. Most studies on freshwater and land snails have focused on their bacterial community, and culturable bacteria have been isolated and analyzed with respect to their potential for producing specific enzymes like cellulases and chitinases. As an example, two cellulolytic strains of the genera *Cellulosimicrobium* and *Streptomyces* were isolated from land snail *Achatina fulica* can be used as a source for biotechnological processes [30]. The freshwater snail *P. acuta* over the last 200 years became cosmopolitan by successfully invading freshwater habitats across the world [20]. Despite the distribution of this freshwater snail, there is no study on antibiotic-producing bacterial species from this organism.

In this study, the Actinobacteria population of the freshwater snail *P. acuta* represented only a minor fraction (2%) of the snail-associated bacterial community. In the freshwater snail *Oncomelania hupensis*, the relative abundance of Actinobacteria was reported to be 14.4% [31]. Based on the 454 pyrosequencing method, the identified bacterial communities in the snails collected from coastal areas showed a higher abundance of Proteobacteria, while Actinobacteria were the more prominent phylum in the mountains of Sichuan and Yunnan Provinces, China [31]. Thus, the bacterial abundance may be affected by many factors, including the niche, diet, physiological states and genetic characteristics of the snail host [32]. In our study, despite the low abundance values of Actinobacteria and, especially, the genus *Streptomyces*, three *Streptomyces* strains (7NS1, 7NS2 and 7NS3) were isolated by using culture-based methods; among them, strain 7NS3 showed strong activity against Gram-positive bacteria. According to the 16S rRNA gene sequence comparisons and whole-genome sequence phylogenetic analysis, 7NS3 showed the highest similarity to *S. seoulensis* NRRL B-24310^T^, respectively. The dDDH analysis suggested that 7NS3 represents a new species of the genus *Streptomyces*, which was deposited as *Streptomyces* sp. DSM 110735 at the DSMZ strain collection. Based on the HR-ESI-MS data of bioactive samples from 7NS3, four compounds were identified, which one of them, named as compounds **3**, matched the characteristics of the compound emycin A. Emycin A was isolated from the cultures of strain *S. griseoincarnatus* DSM 4357 (S1114) in 1989 and was patented due to its novel chemical structure and antiprotozoal activity (U.S. patent 5,100,921). *S. griseoincarnatus* DSM 4357 was isolated from a soil sample collected at Cruz de la Tejeda on the island of Gran Canaria in 1985. No antimicrobial activity has been reported for emycin A so far, which is different from what was found in the current study, where compounds **3** showed antibacterial activity against *B. subtilis* DSM 10^T^ and *S. aureus* Newman. Two other compounds, named compounds **1** and **2**, are suggested to be derivatives of compounds **3** with slightly different chemical structures. The activity also correlated to compounds **4**, which detected as a hexadecenoic acid from the in-house Myxobase database and showed antibacterial activity, especially against *S. aureus.*

Emycin A belongs to the angucycline family, which is the largest group of aromatic polyketide antibiotics. They have an angular tetracyclic framework built up by the action of type II polyketide synthases (PKSs) [25]. Based on the antiSMASH analysis results, the strain 7NS3 harbors a potential angucycline-like BGC, which is suggested to code for the emycin A substance. In order to prove that the identified BGC encodes the biosynthesis of the emycin A substance, it is necessary to construct a respective mutant and perform comparative bioassays and a chemical analysis with the wild-type strain. Furthermore, it would be needed to define gene cluster boundaries of the proposed *ang* gene region by mutational analysis. Such experimental efforts will be part of future studies on 7NS3. Concerning the other biosynthetic gene clusters that were detected by antiSMASH, we could not assign further secondary metabolites to the predicted clusters, with the exception of the emycin A-like compound. This might be because the majority of BGCs in streptomycetes are silent, and thus, the corresponding gene clusters from 7NS3 may not have been expressed under the tested conditions. Overall, this study showed that freshwater snails like *P. acuta* are an under-investigated reservoir for new actinobacterial strains that are able to produce specialized metabolites.

## 4. Materials and Methods

### 4.1. Collection of Snails

In June 2019, five freshwater snails of *P. acuta* were collected from a pond (52°6′42.32″ N, 10°40′46.20″ E) in Remlingen-Semmenstedt, Germany. Snails were transferred to laboratory in two hours and kept in room temperature. After 24 h, snails were washed with sterilized tap water, and their shells were wiped with 70% ethanol. Snails with shells were crushed using a sterile pestle and transferred to 1.5-mL Eppendorf tubes with 500-µL sterile water. The homogenized sample was used for cultivating Actinobacteria and performing DNA extractions.

### 4.2. Microbial Diversity Analysis by High-Throughput V3 Illumina NextSeq Sequencing and Data Analysis

DNA of the whole snail was extracted with the PowerSoil^®^ DNA Isolation Kit and quantified using a nanodrop spectrometer (Thermo Fisher Scientific, Schwerte, NRD, Germany) [33]. The extracted DNA was used to perform a microbial diversity analysis at the Leibniz Institute DSMZ (German Collection of Microorganisms and Cell Cultures GmbH, Braunschweig, Germany). Amplicon preparation of the V3 region of the 16S rRNA gene was performed as described by Bartram et al. with the primer pair 341f and 515r [34]. The quality of the Bartram libraries was checked with a 2100 Bioanalyzer (Agilent Technologies, Santa Clara, CA, USA), and the subsequent sequencing was performed in 150-bp paired end mode on a NextSeq™ 500 (Illumina^®^, San Diego, CA, USA) [34]. The generated sequences were processed with an amplicon analysis pipeline after the quality of the raw reads were checked by FastQC version 0.10.1 (Simon Andrews, Babraham Institute, Cambridgeshire, UK): http://www.bioinformatics.babraham.ac.uk/projects/fastqc/ [35]. After the trimming of the forward and the reverse reads to a length of 130 bp, the raw-sequence data were purified from potential primer dimers by a JAVA program called *DimerFilter*. Fastq-join [36] joined the forward and reverse reads with a 20 percent mismatch and a minimum overlap of 6 bp. FASTA-converted sequence files were subsequently checked with *Uchime* [37] against the Gold database provided by ChimeraSlayer (Robert Edgar, Drive5, Corte Madera, CA, USA) (http://drive5.com/otupipe/gold.tz) and the RDP classifier 2.10.1 [34]. The RDP classifier 2.10.1 [34] with a confidence value of 0.5, which is recommended for short read amplicon data, was employed to perform taxonomic-dependent analyses of the bacterial community [38]. V3 amplicon data was deposited at NCBI SRA (Sequence Read Archives) under accession number PRJNA665462. SRA.

### 4.3. Cultivation of Actinobacteria and 16S rRNA Gene Sanger Sequencing and Phylogenetic Analysis

The amount of 200 µL from the homogenized sample were plated on 5336 agar (10% soluble starch, 1% casein, 0.5% K_2_HPO_4_, 5% MgSO_4_ × 7H_2_O and 20-g/l agar) supplemented with 1% cycloheximide to avoid the growth of fungi [39]. Plates were incubated at 30 °C for up to 10 days. Pure colonies were obtained by dilution plating on 5336 and GYM plates (4% glucose, 4% yeast extract, 10% malt extract, 2% CaCO_3_ and 12-g/L agar) and (≙ DSMZ medium 65) plates. After one week of incubation at 30 °C, genomic DNA was extracted from purified colonies using the Invisorb^®^ Spin Plant Mini Kit following the manufacturer’s instructions. PCR was performed with 1-µL forward 27f (5′-AGAGTTTGATCMTGGCTCAG-3′) and 1-µL reverse primer 1492r (5′-GGTTACCTTGTTACGACTT-3′) and 1 µL of the template DNA (Lane, 1991). Almost a full-length 16S rRNA gene sequence was obtained by using primers 1100f (5′-CAACGAGCGCAACCC-3′), 1100r (5′-GGGTTGCGCTCGTTG-3′) and 518r (5′-CGTATTACCGCGGCTGCTGG-3′) [40]. Obtained sequences were assembled with Bioedit software (version 7.2.6) (https://bioedit.software.informer.com/7.2/). Taxonomic affiliation of the 16S rRNA gene sequences was analyzed with the EzTaxon database (www.ezbiocloud.net) [41]. Pairwise 16S rRNA gene sequence similarities, as well as the maximum-likelihood (ML) and maximum-parsimony (MP) trees, were carried out using the DSMZ phylogenomic pipeline of the genome-to-genome distance calculator server [42] available at http://ggdc.dsmz.de/ [43].

### 4.4. Antimicrobial Test Assay

Three isolated *Streptomyces* strains were cultured in 100 mL of GYM liquid medium for 7 days at 30 °C. Ten milliliters of preculture were inoculated into metabolite medium 5294 (10-g starch, 10-g glucose, 10-g glycerol, 3-g CaCO_3_, 2.5-g corn steep liquor, 2-g peptone and 1-g NaCl) and incubated for 7 days at 28 °C at 160 rpm. The amount of 20 mL from the cell culture was mixed with 20 mL of ethyl acetate and left shaking for one hour at room temperature. The samples were sedimented at 10,052× *g* for 10 min, and the supernatant was transferred into a 50-mL round-bottom flask. The flask was weighed dry and then filled with crude extract. The crude extract was evaporated using a rotary vacuum evaporator, the flask was weighed and the extract residue was dissolved in methanol to adjust to concentration of 1 mg/mL. Crude extracts were screened against nine test microorganisms using the minimal inhibitory concentration method (MIC) [44]. Test bacteria included *Escherichia coli* DSM 1116, *Staphylococcus aureus* (Newman), *Mycobacterium smegmatis* ATCC 700084, *Citrobacter freundii* DSM 30039^T^, *Pseudomonas aeruginosa* PA14 and *Bacillus subtilis* DSM 10^T^, and the tested fungi included *Pichia anomala* DSM 6766, *Mucor hiemalis* DSM 2656^T^ and *Candida albicans* DSM 1665.

MIC was determined by using the final concentration of 1 mg/mL of crude extract with seven 1:2 serial dilution steps in 96-well microplates. Mueller Hinton Broth medium (MHB: 0.5% casein peptone, 0.5% protease peptone, 0.1% meat extract and 0.1% yeast extract, pH 7.0) was used for bacteria and MYC medium (1.0% glucose, 1.0% phytone peptones and 50-mM HEPES (11.9 g/L) for fungi. Twenty microliters of crude extract were added to the first row of wells, which contained 280 µL of bacterial/fungal suspension. After mixing, 150 µL were withdrawn and transferred to the next row of wells. This was repeated sequentially up to the last well. A dilution gradient of 66.6, 33.3, 16.6, 8.3, 4.2, 2.1, 1.0 and 0.52 µg/mL was obtained by discarding 150 µL from the last well. All plates were placed on a microplate shaker incubated for 24 h at 650 rpm at 30 °C for all plates except *M. smegmatis* ATCC 700084 and *E. coli* DSM 1116, which were incubated at 37 °C. The MIC values were defined as the lowest concentration of the tested extract where the growths of the tested microorganisms were not visible to the naked eye. Four biological replicates were tested.

### 4.5. Chemical Analysis of Crude Extracts of 7NS3 Using RP-HPLC and HR-ESI-MS

The crude extract of 7NS3 was fractionated using Reversed-Phase HPLC (RP-HPLC) on an Agilent 1100 HPLC system equipped with XBrigde^®^ (Agilent Technologies, Santa Clara, CA, USA) C-18 3.5 μm, 2.1 mm × 100 mm, Waters column. The fractions (0.15 mL) in the 96-well plates were collected by the HPLC column every 0.5 min and, later, were dried for 45–60 min at 40 °C with heated nitrogen. Afterwards, each well was filled with 150 µL of the *B. subtilis* DSM 10^T^ in MHB medium. HR-ESI-MS (high-resolution electrospray ionization-mass spectrometry) spectra (Bruker Daltonics, Bremen, Germany) were recorded for the strain 7NS3. On an Agilent 1200 system coupled to a DAD (Diode-Array Detection) and a maXis ESI TOF (time of flight) mass spectrometer (Bruker Daltonics, Bremen, Germany), scan range 100–2500 atomic mass unit (amu), rate 2 Hz, capillary voltage 4500 V and drying gas temperature 200 °C using the following HPLC conditions: C18 Acquity UltraPerformance Liquid Chromatography (UPLC) Ethylene Bridged Hybrid (BEH)(Waters) column (2.1 × 50 mm, 1.7 μm), solvent A: H_2_O + 0.1% formic acid and solvent B: acetonitrile (ACN) + 0.1% formic acid, gradient: 5% B for 0.5 min, increasing to 100% B in 20 min, maintaining isocratic conditions at 100% B for 10 min, flow = 0.6 mL/min and UV−Vis detection 200−600 nm. Data processing and analysis was done using data analysis software included in the Compass software from Bruker in order to identify the bioactive target masses. The obtained molecular features were matched against known actinobacterial compounds from the Dictionary of Natural Products (http://dnp.chemnetbase.com/).

### 4.6. Genome Sequencing, dDDH Analysis and BGC Identification

Genomic DNA of 7NS3 was extracted following the protocol described by Jin et al. [45]. Libraries for whole-genome sequencing on the Illumina platform were prepared from extracted genomic DNA applying the Nextera XT DNA Library Preparation Kit (Illumina, San Diego, CA, USA) with modifications [34]. Samples were sequenced on the Illumina NextSeq™ 500. Hereby, 5,155,283 Mio paired-end reads of 2 × 151 bp were obtained. Short read genome assembly was performed using SpaDES 3.14 using default parameters [46]. The whole-genome sequence was submitted to the NCBI GenBank under accession number JACYHF000000000, applying the NCBI Prokaryotic Annotation Pipeline PGAP (pan-genome analysis pipeline).

Whole-genome phylogeny was generated using the TYGS server (https://tygs.dsmz.de) [24]. In addition, digital DNA–DNA hybridization (dDDH) values between the genome sequences of 7NS3 and *S. seoulensis* NRRL B-24310^T^ were calculated using the Genome-to-Genome Distance Calculator GGDC 2.1 (http://ggdc.dsmz.de) [43]. To assess the potential of 7NS3 for secondary metabolite biosynthesis, an antiSMASH analysis was carried out with version 5.0 (https://antismash.secondarymetabolites.org) [47]. 

## Figures and Tables

**Table 1:** Minimum inhibitory concentration ratios (µg/mL) with 1 mg/mL of extracts from strains 7NS1, 7NS2 and 7NS3 against the tested microorganisms.

Scheme 10.	*S. aureus* Newman	*B. subtilis* DSM 10^T^	*M. smegmatis* ATCC 700084	*E. coli* DSM 1116	*P. aeruginosa* PA14	*C. freundii* DSM 30039^T^	*C. albicans* DSM 1665	*P. anomala* DSM 6766	*M. hiemalis* DSM 2656^T^
7NS1	>66.6	16.6	>66.6	-	-	-	>66.6	-	16.6
7NS2	16.6	16.6	>66.6	-	-	-	-	-	>66.6
7NS3	8.3	0.52	>66.6	-	-	-	-	-	>66.6

(-): no inhibition.

## Data Availability

The data presented in this study are available in supplementary material.

## Supplementary Materials

The following are available online at https://www.mdpi.com/2079-6382/10/1/22/s1, Figure S1: HPLC fractionation result in 96-well plate (top), time map (middle) and HPLC chromatogram (bottom), Figure S2: HR-ESI-MS data of four targeted peaks, UV chromatogram (left) and total ion current (TIC) (right), Figure S3: Chemical structure and characteristics of emycin A (Adopted from Dictionary of Natural Products), Figure S4: List of predicted secondary metabolite gene clusters for strain 7NS3 identified by analysis of the 7NS3 genome sequence with the bioinformatic tool antiSMASH 5.0, Figure S5: Schematic presentation of BGC region 6.1 of strain 7NS3. Table S1: Number of reads assigned to actinobacterial families, Table S2: 7NS3 region 6.1 genes and their deduced functions.
