# Supplementary material for: Angucycline-like Aromatic Polyketide from a Novel Streptomyces Species Reveals Freshwater Snail Physa acuta as Underexplored Reservoir for Antibiotic-Producing Actinomycetes
Source: Antibiotics (Basel). 2020 Dec 29;10(1):22. doi: 10.3390/antibiotics10010022 (PMC7823578; doi:10.3390/antibiotics10010022)
Supplement: Supplementary file 1 [file antibiotics-10-00022-s001.pdf]

antibiotics

MDPI

Article

Angucycline-like Aromatic Polyketide from a Novel *Streptomyces* Species Reveals Freshwater Snail *Physa acuta* as Underexplored Reservoir for Antibiotic-producing Actinomycetes

Nasim Safaei [1], Yvonne Mast [2], Michael Steinert [3], Katharina Huber [2], Boyke Bunk [2] and Joachim Wink [1,*]

1. Department of microbial Strain Collection, Helmholtz Centre for Infection Research, Inhoffenstrasse 7, D-38124 Braunschweig, Germany; Nasim.safaei@helmholtz-hzi.de (N.S.); Joachim.wink@helmholtz-hzi.de (J.W.)
2. Leibniz Institute DSMZ, German Center for Infection Research (DZIF), Inhoffenstrasse 7, D-38124 Braunschweig, Germany; Yvonne.mast@dsmz.de (Y.M.); Katharina.huber@dsmz.de (K.H.); Boyke.bunk@dsmz.de (B.B.)
3. Technical University of Braunschweig, Spielmannstr. 7, 38106 Braunschweig, Germany; m.steinert@tu-bs.de

* Correspondence: Joachim.wink@helmholtz-hzi.de; Tel.: +49-531-6181-4223

Supplementary Materials:

Table 1. Number of reads assigned to actinobacterial families.

Family of Actinobacteria	Read counts
Kineosporiaceae	1688
Microbacteriaceae	1298
Acidimicrobiaceae	1154
Nocardioidaceae	622
Nocardiaceae	391
Micromonosporaceae	339
Coriobacteriaceae	220
Propionibacteriaceae	146
Intrasporangiaceae	95
Pseudonocardiaceae	79
Tsukamurellaceae	50
Thermoleophilaceae	26
Corynebacteriaceae	21
Streptosporangiaceae	21
Micrococcaceae	20
Mycobacteriaceae	17
Nakamurellaceae	13
Cellulomonadaceae	13
Geodermatophilaceae	10
Nocardiopsaceae	9
Catenulisporaceae	7
Thermomonosporaceae	6
Solirubrobacteraceae	5
Conexibacteraceae	5
Rubrobacteraceae	5
Bifidobacteriaceae	4
Iamiaceae	4
Streptomycetaceae	4
Gaiellaceae	3
Patulibacteraceae	1

**Total**	**6276**

A

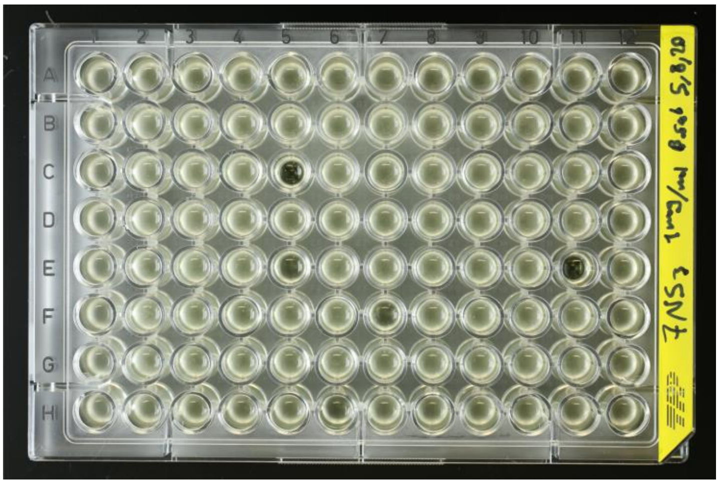

Time map

	1	2	3	4	5	6	7	8	9	10	11	12
A		7.5		15.5		23.5		31.5		40.0		
B	0.5		8.5		16.5		24.5		32.5			
C		6.5		14.5		22.5		30.5		38.5		
D	1.5		9.5		17.5		25.5		33.5			
E		5.5		13.5		21.5		29.5		37.5		
F	2.5		10.5		18.5		26.5		34.5			
G		4.5		12.5		20.5		28.5		36.5		
H	3.5		11.5		19.5		27.5		35.5			

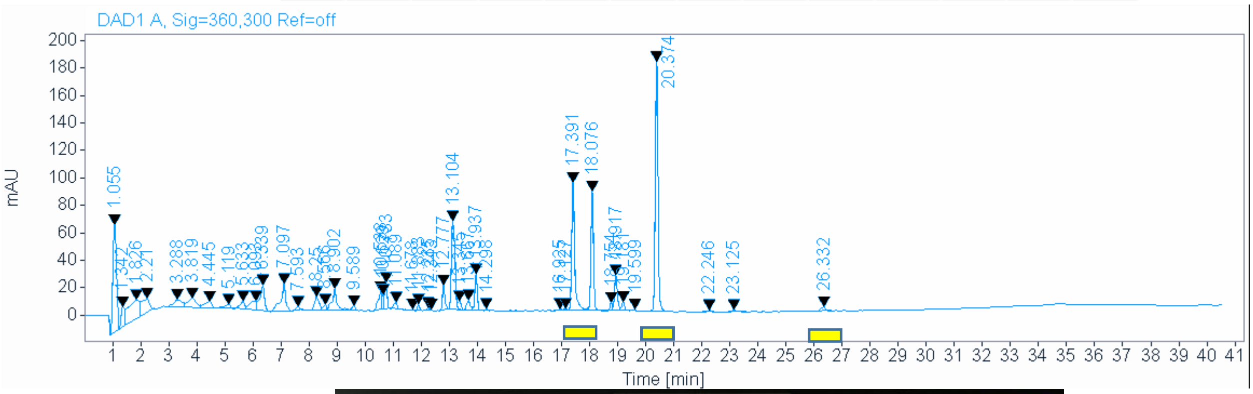

B

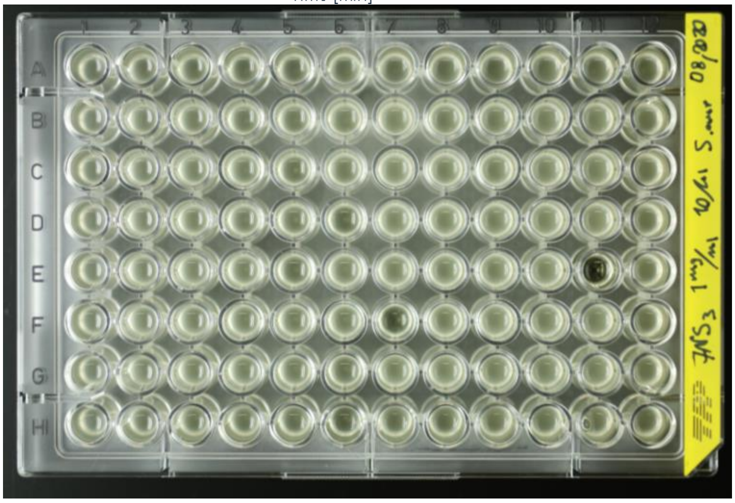

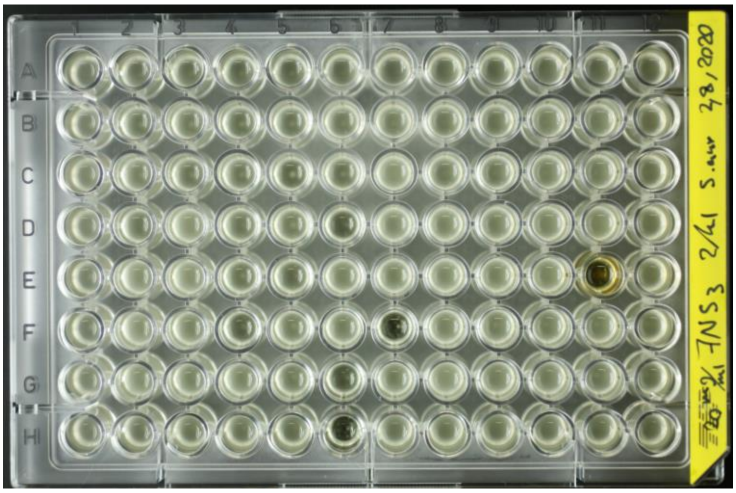

Time map

	1	2	3	4	5	6	7	8	9	10	11	12
A		7.5		15.5		23.5		31.5		40.0		
B	0.5		8.5		16.5		24.5		32.5			
C		6.5		14.5		22.5		30.5		38.5		
D	1.5		9.5		17.5		25.5		33.5			
E		5.5		13.5		21.5		29.5		37.5		
F	2.5		10.5		18.5		26.5		34.5			
G		4.5		12.5		20.5		28.5		36.5		
H	3.5		11.5		19.5		27.5		35.5			

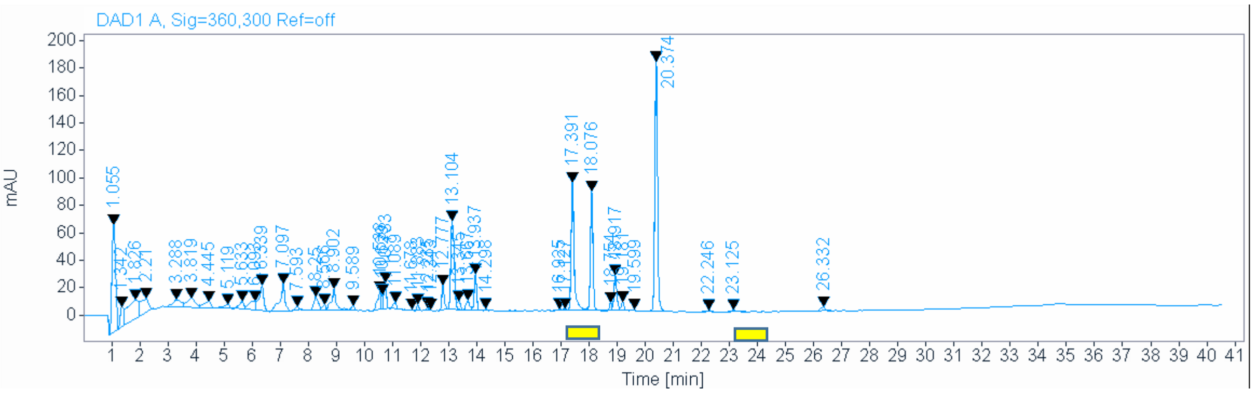

Figure 1. HPLC fractionation result in 96-well plate (top), time map (middle) and HPLC chromatogram (bottom); A) Micro-fractionation of crude extract (final concentration of 1mg/ml) against *B. subtilis* DSM 10^T, fractions highlighted in time map (fractions were collected by the HPLC column every 0.5 min) containing corresponding peaks at retention time (t_R) = 17, 18, 20 and 26.5 min. The corresponding peaks highlighted in HPLC chromatogram. B) Micro-fractionation of crude extract (two 96-well plates with final concentration of 1mg/ml (top) and 20mg/ml (bottom)) against *S. aureus* Newman, fractions containing corresponding peaks at t_R = 20 and 26.5 min which are highlighted in HPLC chromatogram. 10µl of crude extract was used as control in well 11E.

A

1, 10.39 min

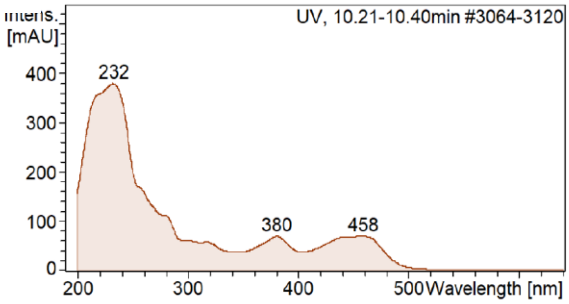

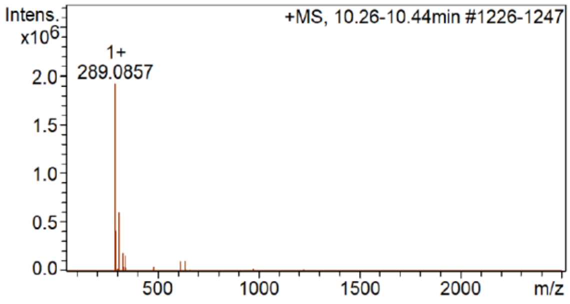

#	Wavelength	Intensity
0	232	379.3
1	380	71.0
2	458	71.4

#	m/z	I	I %
1	289.0857	1923677	100.0
2	290.0891	412982	21.5
3	291.0969	80123	4.2
4	307.0963	609077	31.7
5	308.0996	129880	6.8
6	329.0783	184341	9.6
7	340.1541	156711	8.1
8	613.1858	97475	5.1
9	635.1675	102526	5.3
10	636.1709	43746	2.3

B

2, 10.49 min

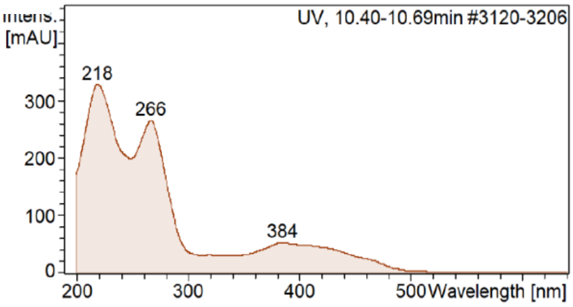

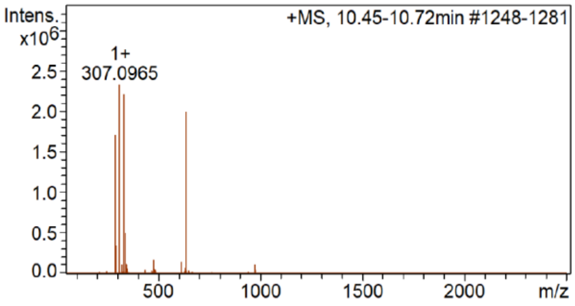

#	Wavelength	Intensity
0	218	327.6
1	266	265.0
2	384	54.4

#	m/z	I	I %
1	289.0856	1701919	73.0
2	290.0890	350787	15.0
3	307.0965	2331032	100.0
4	308.0995	1275209	54.7
5	329.0783	2200823	94.4
6	330.0816	580865	24.9
7	340.1540	502333	21.5
8	635.1675	1990029	85.4
9	636.1708	889244	38.1
10	637.1740	208982	9.0

C

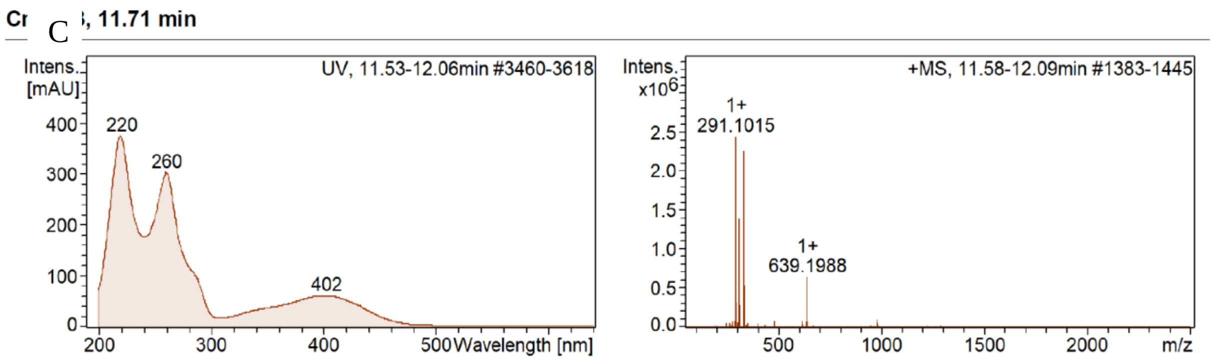

#	Wavelength	Intensity
0	220	374.3
1	260	301.9
2	402	62.9

#	m/z	I	I %
1	291.1015	2438167	100.0
2	292.1048	2235877	91.7
3	293.1077	328180	13.5
4	309.1119	1385564	56.8
5	310.1153	286650	11.8
6	329.0783	330970	13.6
7	331.0940	2246167	92.1
8	332.0973	537095	22.0
9	639.1988	641523	26.3
10	640.2024	266093	10.9

D

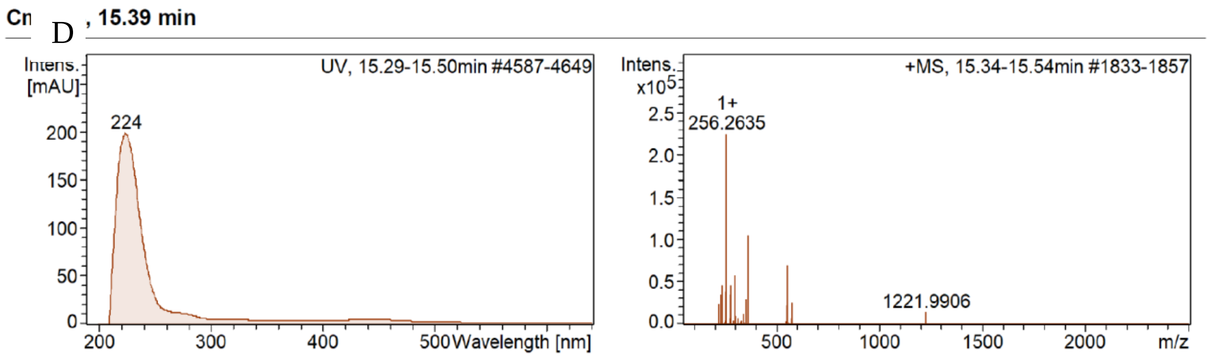

#	Wavelength	Intensity
0	224	198.1

#	m/z	I	I %
1	229.2161	35358	15.7
2	237.2211	46302	20.6
3	255.2319	39040	17.4
4	256.2635	224808	100.0
5	257.2669	40787	18.1
6	277.2138	35713	15.9
7	278.2454	46116	20.5
8	299.1957	57674	25.7
9	365.2663	105211	46.8
10	555.1803	69752	31.0

Figure 2. HR-ESI-MS data of four targeted peaks, UV chromatogram (left) and total ion current (TIC) (right). A) Peak 1 at t_R = 10.39 min: m/z 289.0857 MH^+, UV absorption bands at 232, 380 and 458 nm; B) Peak 2 at t_R = 10.49 min: m/z 307.0965 MH^+, UV absorption bands at 218, 266 and 384 nm; C) Peak 3 at t_R = 11.71 min: m/z 309.1119 MH^+, UV absorption bands at 220, 260 and 402 nm; D) Peak 4 at t_R = 15.39 min: m/z 256.2635 MH^+, UV absorption band at 224 nm.

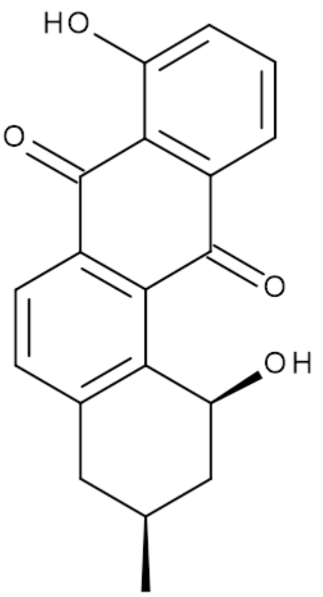

Derivative: 4-Deoxy, *O*-de-Me

Synonym(s): Emycin A.

CRC Number: DOL56-W

CAS Registry Number: 127414-87-3

Type of Compound Code(s): D.C.04500 V.C.04500 Z.B.70000

Molecular Formula: $C_{19}H_{16}O_4$

Molecular Weight: 308.333

Accurate Mass: 308.10486

Percentage Composition: C 74.01%; H 5.23%; O 20.76%

Biological Source: Prod. by *Streptomyces* sp. DSM 4357

Solubility: Sol. MeOH, DMSO, dioxan, Me_2CO, $CHCl_3$; poorly sol. H_2O, hexane

UV: [neutral]λ_{max}217;260;402 (MeOH)

Smiles: CC1CC(O)c2c(C1)ccc1C(=O)c3c(O)cccc3C(=O)c21

InChi Key: InChIKey=OBCFJTYDPHCLMZ-UHFFFAOYSA-N

InChi: InChI=1S/C19H16O4/c1-9-7-10-5-6-12-17(15(10)14(21)8-9)19(23)11-3-2-4-13(20)16(11)18(12)22/h2-6,9,14,20-21H,7-8H2,1H3

Figure 3. Chemical structure and characteristics of emycin A (Adopted from Dictionary of Natural Products).

Region	Type	From	To	Most similar known cluster		Similarity
Region 2.1	NRPS-like	72	31,567	stenothricin	NRP:Cyclic depsipeptide	13%
Region 2.2	T3PKS	210,892	250,828	alkylresorcinol	Polyketide	100%
Region 2.3	NRPS, T3PKS	333,972	434,416	scabichelin	NRP	100%
Region 3.1	lassopeptide	44,881	67,030			
Region 3.2	lanthipeptide, bacteriocin	108,242	135,416	informatipeptin	RiPP:Lanthipeptide	100%
Region 5.1	siderophore	56,673	68,634			
Region 5.2	NRPS, T1PKS, terpene	181,761	279,623	naphthyridinomycin	NRP	7%
Region 6.1	T2PKS, siderophore	159,682	232,476	lugdunomycin	Polyketide	44%
Region 6.2	terpene	312,188	334,347	geosmin	Terpene	100%
Region 6.3	bacteriocin	344,596	355,909			
Region 7.1	terpene	57,132	78,214	albaflavenone	Terpene	100%
Region 8.1	terpene	228,203	249,093	BD-12	NRP	17%
Region 10.1	siderophore	61,405	73,174	desferrioxamin B / desferrioxamine E	Other	66%
Region 14.1	ectoine	63,866	74,270	ectoine	Other	100%
Region 15.1	T2PKS	58,891	131,396	spore pigment	Polyketide	83%
Region 17.1	NRPS	41,793	96,219	ibomycin	Polyketide	7%
Region 18.1	melanin	123,893	134,438	melanin	Other	60%
Region 24.1	LAP, thiopeptide	47,415	82,274	thiotetroamide	Polyketide	11%
Region 40.1	terpene	1	13,551	hopene	Terpene	61%

Figure 4. List of predicted secondary metabolite gene clusters for strain 7NS3 identified by analysis of the 7NS3 genome sequence with the bioinformatic tool antiSMASH 5.0. The term "region" describes the genome sequence area on which the biosynthesis gene cluster is localized [47].

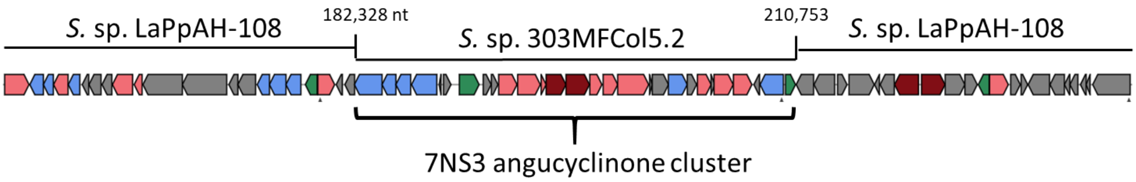

Figure 5. Schematic presentation of BGC region 6.1 of strain 7NS3. Black lines indicate similarities to similar gene regions in *Streptomyces* sp. 303MFCol5.2 and *Streptomyces* sp. LaPpAH-108, respectively. Potential angucycline-type BGC is highlighted by a bracket. Individual genes are shown as arrows and are marked with the antiSMASH-specific color-code [47]: core biosynthetic genes (red), additional biosynthetic genes (red), transport-related genes (blue), regulatory genes (green), other genes (grey).

Table 2. 7NS3 region 6.1 genes and their deduced functions.

Gene-No.	Predicted function	ID/SM†	Matched strain	Accession number
IF655_12470	ABC transporter ATP-binding protein	69/75	*S.* sp. 303MFCol5.2	WP_020126666
IF655_12475	ABC transporter permease	81/89	*S.* sp. 303MFCol5.2	WP_020130958
IF655_12480	ABC transporter permease	85/92	*S.* sp. 303MFCol5.2	WP_020130959
IF655_12485	ABC transporter substrate-binding protein	78/85	*S.* sp. 303MFCol5.2	WP_037735983
IF655_12490	Nuclear transport factor 2 family protein	92/95	*S.* sp. 303MFCol5.2	WP_020130962
IF655_12495	LuxR family transcriptional regulator	81/87	*S.* sp. 303MFCol5.2	WP_159088283
IF655_12500	Nuclear transport factor 2 family protein	87/92	*S.* sp. 303MFCol5.2	WP_020130964
IF655_12505	MarR family transcriptional regulator	81/87	*S.* sp. 303MFCol5.2	WP_159088284
IF655_12510	Acyl-CoA dehydrogenase family protein	88/92	*S.* sp. 303MFCol5.2	WP_020130966
IF655_12515	FAD-dependent monooxygenase	85/89	*S.* sp. 303MFCol5.2	WP_020130967
IF655_12520	TcmI family type II polyketide cyclase	85/89	*S.* sp. 303MFCol5.2	WP_020130968
IF655_12525	Beta-ketoacyl-[acyl-carrier-protein] synthase family protein	92/95	*S.* sp. 303MFCol5.2	WP_020130969
IF655_12530	Ketosynthase chain-length factor	90/92	*S.* sp. 303MFCol5.2	WP_020130970
IF655_12535	3-oxoacyl-ACP reductase FabG	92/96	*S.* sp. 303MFCol5.2	WP_020130972
IF655_12540	Aromatase/cyclase	90/93	*S.* sp. 303MFCol5.2	WP_020130973
IF655_12545	SDR family oxidoreductase	83/87	*S.* sp. 303MFCol5.2	WP_020130974
IF655_12550	Hypothetical protein	62/63	*S.* sp. 303MFCol5.2	WP_020130975
IF655_12555	LLM class flavin-dependent oxidoreductase	91/93	*S.* sp. 303MFCol5.2	WP_020130976
IF655_12560	MFS transporter	81/86	*S.* sp. 303MFCol5.2	WP_078615849
IF655_12565	NAD(P)H-dependent oxidoreductase	88/93	*S.* sp. 303MFCol5.2	WP_179534090

IF655_12570	TIGR03621 family F420-dependent LLM class oxidoreductase	88/92	*S.* sp. 303MFCol5.2	WP_020130979
IF655_12575	Ferredoxin	77/83	*S.* sp. 303MFCol5.2	WP_028807030
IF655_12580	FAD-dependent oxidoreductase	72/80	*S.* sp. 303MFCol5.2	WP_020130981
IF655_12585	cytochrome P450	86/91	*S.* sp. 303MFCol5.2	WP_108222034
IF655_12590	helix-turn-helix domain-containing protein	85/89	*S.* sp. 303MFCol5.2	WP_020130983
IF655_12595	DHA2 family efflux MFS transporter permease subunit	85/88	*S.* sp. 303MFCol5.2	WP_020130984
IF655_12600	TetR/AcrR family transcriptional regulator	88/91	*S.* sp. 303MFCol5.2	WP_020130985

ORF, open reading frame; †ID/SM, % identity/similarity of amino acid sequences *S.* sp. 303MFCol5.2, *Streptomyces* sp. 303MFCol5.2.
